# Ov2 is a modulator of OvHV-2 RTA mediated gene expression

**DOI:** 10.1007/s11259-019-09748-w

**Published:** 2019-03-21

**Authors:** Inga Dry, Katie Nightingale, Jack Ferguson, John Hopkins, Robert Dalziel

**Affiliations:** 1 The Roslin Institute & R(D)SVS, University of Edinburgh, Edinburgh, Midlothian, EH25 9RG UK; 2 Present Address: Cambridge Institute of Medical Research, Hills Road, Cambridge, CB2 0XY UK; 3 Present Address: Institute of Cancer and Genomic Sciences, IBR West, College of Medical and Dental sciences, University of Birmingham, Edgebaston, B15 2TT UK

**Keywords:** Ovine herpesvirus-2, Ov2, Gene expression

## Abstract

Ovine herpesvirus-2 (OvHV-2) is the causative agent of the sheep-associated form of malignant catarrhal fever, a usually fatal lymphoproliferative disease of bison, deer and cattle. Malignant catarrhal fever is a major cause of cattle loss in Africa with approximately 7% affected annually; and in North America has significant impact on bison farming. Research into the mechanisms by which OvHV-2 induces disease in susceptible species has been hampered by a lack of a cell culture system for the virus. Ov2 is a bZIP protein encoded by OvHV-2. Proteins with bZIP domains in other herpesviruses, such as the Kaposi’s sarcoma-associated herpesvirus K8 protein and the BZLF1 protein of Epstein-Barr virus are known to play important roles in lytic virus replication. Using a reporter based system, we demonstrate that Ov2 can modulate the activity of the major virus transactivator (Replication and Transcriptional Activator protein, RTA) to 1) drive expression of viral genes predicted to be required for efficient reactivation of the virus, including ORF49; and 2) differentially regulate the expression of the two virus encoded Bcl-2 homologues Ov4.5 and Ov9.

## Introduction

Malignant catarrhal fever (MCF) is a usually fatal disease of cattle, deer, bison and other ruminants caused by viruses in the genus *Macavirus* of the subfamily *Gammaherpesvirinae* (McGeoch et al. 2006). MCF is characterised by sudden onset of fever followed by lymphadenopathy, leucocytosis, severe congestion and necrosis and erosion of the oral, conjunctival and nasal muscosæ (Russell et al. 2009). The two most common forms of MCF detected are the sheep associated form of the disease caused by Ovine herpesvirus 2 (OvHV-2) and the wildebeest associated form of disease caused by Alcelaphine herpesvirus 1 (AlHV-1). OvHV-2 and AlHV-1 subclinically-infect and establish a lifelong latent infection, within lymphocytes of sheep or wildebeest, making these species reservoir hosts. Reactivation of these viruses, and the other *Macaviruses* from latency within reservoir populations, resulting in shedding of the virus in nasal secretions, poses an infection risk to MCF susceptible species, such as cattle, bison and deer (O'Toole and Li 2014).

Lytic replication of OvHV-2 in sheep, the reservoir of the disease, is reliably detected only in the nasal turbinates and the lung (Li et al. 2014). Furthermore, shedding of OvHV-2 in nasal secretions from both adolescent and adult sheep appears to occur in short, sharp bursts that suggest a relatively tightly controlled reactivation process (Li et al. 2014).

The regulation of the process of reactivation from latency, and the viral proteins that drive the lytic cycle of OvHV-2 are not yet clearly defined. Evidence from the study of Kaposi’s sarcoma-associated herpesvirus (KSHV) and murine gamma herpesvirus 68 (MHV-68) has indicated, that for these viruses, expression of the replication and transcription activator (RTA; encoded by ORF50), is sufficient to induce lytic replication (Gradoville et al. 2000; Wu et al. 2000). In contrast, induction of the Epstein Barr virus (EBV) lytic cycle requires the expression of BRLF1, the EBV homolog of RTA, and BZLF1, a virally encoded bZIP domain containing protein (Zalani et al. 1996) (Liu and Speck 2003; Speck et al. 1997). In EBV, a further virus protein, BRRF1, has been identified as an enhancer of lytic replication (Hong et al. 2004).

Analysis of the genome of OvHV-2 determined that OvHV-2 encodes homologs of RTA and BRRF1(Hart et al. 2007). Moreover, genomic analysis also identified within the genome of OvHV-2 the presence of an open reading frame predicted to code for a basic leucine zipper (bZIP) family protein homologue (Hart et al. 2007). The function of this bZIP protein homolog, Ov2, in OvHV-2 biology is not well understood. The genome of AlHV-1, a related *Macavirus*, contains a positional homologue of Ov2, termed A2, that also contains a bZIP domain (Ensser et al. 1997). In a rabbit model of MCF, up regulation of cellular apoptosis pathways was observed following infection with a virus carrying a deletion of the A2 gene (Parameswaran et al. 2014).

Evidence from MHV-68 and KSHV suggests a role for viral Bcl-2 homologs in both the establishment of chronic infections and in reactivation of the virus from latency (Coleman et al. 2014; Hwang et al. 2009; Gelgor et al. 2015). OvHV-2 encodes two Bcl-2 homologs, ORFs Ov4.5 and Ov9 (Hart et al. 2007) and in light of the increased level of apoptosis seen in A2 deleted virus infected cells we hypothesised that Ov2 may influence the expression of these genes.

This study aimed to investigate the whether Ov2 plays a role in the control of virus gene expression. Due to the lack of a productive cell culture system for OvHV-2, a luciferase reporter-based promoter assay was utilised to demonstrate that Ov2 is capable of modulating the viral replication and transcription activator (RTA; encoded by ORF50) to repress or activate expression of virus encoded genes including ORF49, Ov4.5, Ov9, ORF50 and ORF57.

## Methods

### Cell culture

Baby hamster kidney cells (BHK-21) were cultured in Dulbecco’s Modified Eagle Medium (Invitrogen) supplemented with 10% (*v*/v) FCS and 1% (v/v) penicillin-streptomycin-glutamine (Invitrogen) and incubated at 37 °C, 5% CO_2_.

### Cloning of OvHV-2 gene and promoter sequences

DNA was extracted from OvHV-2 positive BJ1035 cells (Schock et al. 1998) using the DNA Blood and Tissue kit (Qiagen, UK).

The OvHV-2 ORF50 gene was cloned in a multi-step process. Each PCR reaction contained 1 unit HotStarTaq *Plus* DNA polymerase (Qiagen), 50 ng BJ1035 DNA, 200 μM dNTPS and 12 pmols of each primer in a final reaction volume of 20 μl . To allow us to confirm expression of the RTA protein the OvHV-2 ORF50 gene sequence was cloned such that the RTA was expressed as a fusion protein with a Haemagglutinin tag. All PCR reactions to amplify the ORF50 gene were carried out using cycling conditions consisting of an initial denaturation 5 min at 94 °C, 30 cycles of 94 °C for 30 s, 50 °C for 1 min and 72 °C for 3 min 15 s and a final extension of 72 °C for 7 min. The first fragment was generated using the primer sets ORF50 Fwd and ORF50R1rev and the second fragment was amplified using the primers ORF50 R2 and ORF50HA rev. (All primers are shown in Table 1). Full-length product was generated by annealing and amplifying fragments 1 and 2 in the presence of ORF50 Fwd and HA rev primers.

**Table 1:** Oligonucleotide primers used to amplify and clone genes encoding Ov2 and RTA and viral promoters. OvHV-2 RTA is encoded by the ORF50 gene

Primer name	Primer Sequence	Use
ORF50 Fwd	GGATCCACCATGAGTGGCAAAAGACCCTC	For cloning into pcDNA3.1+
ORF50HA rev	AGCGTAATCTGGAACATCGTATGGGTACTGAAACCCTGAGGAGTTG	Amplification of ORF50 to delete ORF49
ORF50R1	TCGTCTAGGCATATTACCTTGGAAATACTCTTCTTCTTTGGGGGTCCAT	Amplification of ORF50 to delete ORF49
ORF50 R2	CATGGACCCCCAAAGAAGAAGAGTATTTCCAGGTAATATGC	For cloning into pcDNA3.1+
HA rev	TTAAGCGTAATCTGGAACATCGTATGGGTA	Amplifying a HA tag + addition of a stop codon
ORF57pS	GGTACCCACTAGCTTCCCCGCCGG	Cloning into pGL3basic
ORF57pAS	ACGCGTCCTTCAACGGTCCGGTTC	Cloning into pGL3basic
ORF50pS	GGTACCTGTAGATCTCTTACTGAGTG	Cloning into pGL3basic
ORF50pAS	ACGCGTGGTCCATGCTGACTGTGGTC	Cloning into pGL3basic
ORF49pS	GGTACCTAC AAA CAG GAT GGG AAG	Cloning into pGL3basic
ORF49pAS	ACG CGT TTG TCT GGG TGC TCG TCG	Cloning into pGL3basic
ORF6pS	GGTACCCAACGAGGAGGTCCGC	Cloning into pGL3basic
ORF6pAS	ACGCGTGCCTTGGACCCGATATTATC	Cloning into pGL3basic
ORF25pS	GGTACC GCAGTTCTTGGGGCTCC	Cloning into pGL3basic
ORF25pAS	ACGCGTTCTACGGCTGTGTGGGGAAG	Cloning into pGL3basic
Ov9pS	GGTACCGGTATAAGGGTGCTTTAAG	Cloning into pGL3basic
Ov9pAS	ACGCGTGTCCAGTGGCTCCCAGTG	Cloning into pGL3basic
Ov4.5pS	GGTACCAGTCCCGACGCCCTCCTG	Cloning into pGL3basic
Ov4.5pAS	ACGCGTGGCCGCATACTGTGTGGTAG	Cloning into pGL3basic

PCR products were purified using a QIAquick PCR purification kit (Qiagen) and cloned into PCR®2.1 TOPO vector (Life Technologies, UK). Sequencing by GATC (Cologne, Germany) confirmed the absence of any additional mutations to ORF50. The ORF50 open reading frame was subcloned in frame into the expression vector pcDNA3.1+ (Life Technologies) using *Bam*H1 and *Eco*R1. The cloning of Ov2 into pcDNA3.1+ is described in detail in the accompanying paper (Nightingale et al. 2019).

OvHv-2 gene promotor sequences primers were designed to amplify approximately 1000 bp upstream and 50 bp downstream of the initiation codon of each gene of interest as marked on the published OvHv-2 genome (Genbank accession: AY839756.1). Promoters were amplified from BJ1035 DNA. Each PCR reaction contained 1 unit HotStarTaq *Plus* DNA polymerase (Qiagen), 8 pmols of primers, 50 ng DNA and 200 μM dNTPS in a final reaction volume of 20 μl.The cycling conditions used for amplification were an initial denaturation of 5 mins at 95 °C, followed by 30 cycles of 95 °C for 30 s, 58 °C for 1 min and 72 °C for 1 min, with a final extension of 72 °C for 7 min. Each putative promoter was cloned into pGL3basic (Promega) using *Kpn1* and *Mlu1* restriction sites. Promoter constructs are named ORFXXp: e.g. ORF50p. All primers were synthesised by Sigma-Aldrich.

### Luciferase promoter assays

DNA was transfected, using Lipofectamine 2000 (Invitrogen), into BHK21 cells seeded at a density of 2 × 10^5^ cells per well 24 h prior to transfection. For each promoter assay 40 ng pRL-SV40 (Renilla luciferase (RL) transfection control) was transfected alongside 820 ng of the specific promoter construct expressing Firefly luciferase (FL). Equivalent amounts of expression constructs were used per transfection and empty pcDNA3.1+ was used as carrier DNA, where necessary to take the final DNA concentration per transfection to 2.5 μg. Cells were washed once in PBS 48 h after transfection, and lysed with 1 x Passive Lysis Buffer (Promega). Luciferase signals were detected using a 96 Microplate Glomax Luminometer, in conjunction with the Dual-Luciferase Reporter Assay system (both Promega). Relative light units for each promoter tested was calculated by: firefly luciferase (FL) reading/*Renilla* luciferase (RL) reading. Where stated in the text fold change was calculated ([FL/RL] plus expression construct/ [FL/RL] with empty vector).

### Statistical analysis

Statistical analysis was performed using Minitab 17 software. Differences between groups were analysed using a general linear model followed by Tukey’s post-hoc test. *P*-values represent results from the post-hoc test.

### Illustrations

Illustrations were generated using GraphPad Prism.

## Results

### Regulation of viral gene expression by Ov2 protein

No tissue culture system is available to study the role of specific virus encoded proteins in OvHV-2 replication therefore to investigate if Ov2 either alone, or in concert with the OvHV-2 RTA, could activate or repress transcription from a variety of viral promoters, a luciferase based reporter gene system was utilised. This approach was used successfully to characterise the transcriptional control of the AlHV-1 RTA (Frame and Dalziel 2008).

**Fig. 1:**
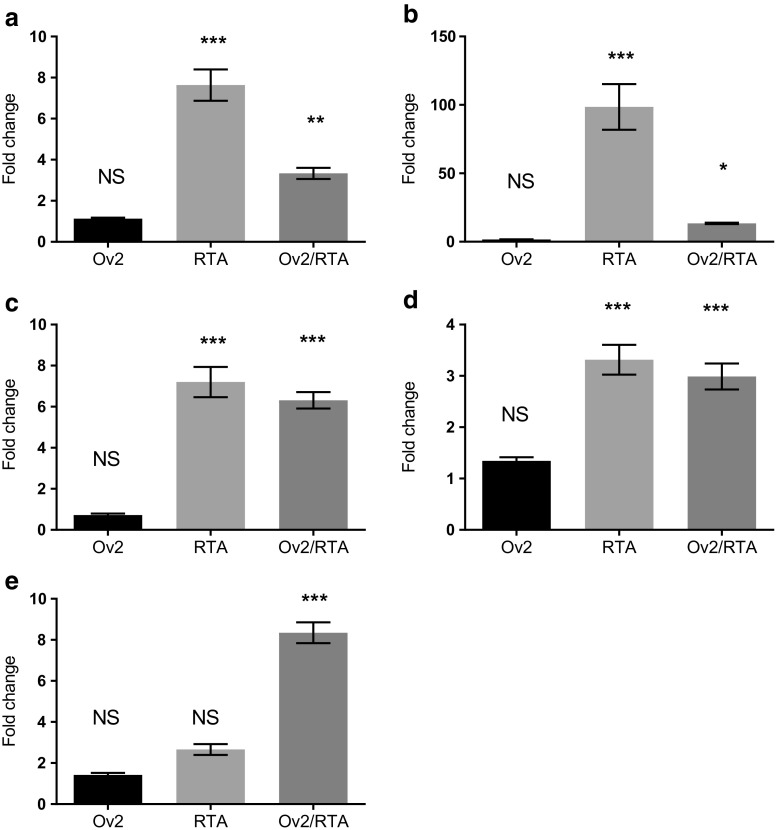
**Response of OvHv-2 promoters to Ov2 protein alone, RTA alone or Ov2 and RTA in combination.** BHK cells were transfected with promoter constructs (**a**) ORF50p (**b**) ORF57p (**c**) ORF6p (**d**) ORF25p (**e**) ORF49p, along with Ov2 or RTA expression constructs as indicated. All samples were also transfected with the pRLSV40 plasmid as an internal control. Expression of luciferase activity was detected 48 h post transfection. The graph represents the results from three independent experiments. Transfections were carried out in quadruplicate. Each bar represents the average fold change over basal promoter activity. NS; not significant * = *p* ≤ 0.05, ** = *p* ≤ 0.01, *** = *p* ≤ 0.0001

**Fig. 2:**
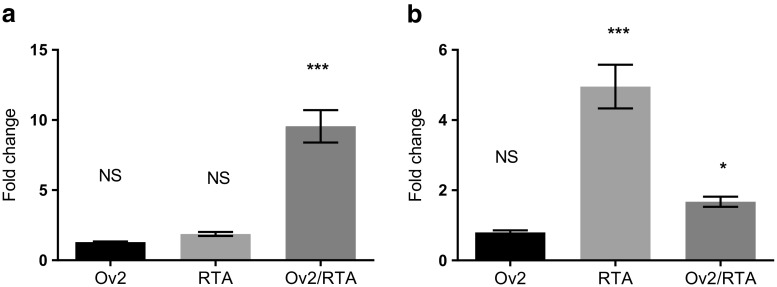
**Ov2 differentially regulates Ov9 and Ov4.5.** BHK cells were transfected with promoter constructs (**a**) Ov9p (**b**) Ov4.5p. Along with Ov2 or RTA expression constructs indicated. All samples were also transfected with the pRLSV40 plasmid as an internal control. Expression of luciferase activity was detected 48 h post transfection. The graph represents the results from three independent experiments. Transfections were performed in quadruplicate. Each bar represents the average fold change over basal promoter activity. NS;not significant* = p ≤ 0.05, *** = p ≤ 0.0001

Expression of Ov2 protein and RTA, from our constructs, was confirmed by Immunofluorescence using a custom OV2 specific Ab (see accompanying paper; Nightingale et al. 2019) or a commercial antibody to the HA tag expressed on the RTA construct (data not shown). Cell viability assays confirmed that there was no differences in viability between cells that were single or double-transfected (data not shown). The ability of Ov2 protein and RTA to stimulate transcription from each promoter independently and co-operatively was tested. Individually, Ov2 protein showed no ability to stimulate or repress any of the promoters tested (*p* ≤ 0.32 to 0.99). In contrast, the presence of RTA alone was found to be sufficient to stimulate transcription from ORF25p, ORF6p, ORF50p (RTA), ORF57p (Fig. 1) and Ov4.5p (Fig. 2). However it was also noted that RTA, by itself, was unable to stimulate transcription from either ORF49p (*p* ≤ 0.06; Fig. 1) or Ov9p (*p* ≤ 0.80; Fig. 2) above basal levels.

Co-expression of Ov2 protein and RTA resulted in approximately 50 and 90% reductions in the expression of the luciferase reporter gene observed from ORF50p (RTA) and ORF57p driven constructs respectively, compared with expression of RTA alone (*p* ≤ 0.001; Fig. 1). No significant difference was observed on the expression from ORF6p (*p* ≤ 0.99) or ORF25p (*p* ≤ 0.84), compared to RTA alone, when Ov2 was co-expressed with RTA (Fig. 1). In contrast, co-expression of Ov2 protein and RTA led to an approximately 8 fold stimulation of transcription from the ORF49 promoter (*p* ≤ 0.0001; Fig. 1) and an approximately 5 fold stimulation of transcription of the Ov9 promoter (p ≤ 0.0001; Fig. 2). Moreover, co-expression of Ov2 protein with RTA was found to inhibit RTA-mediated stimulation of transcription from the Ov4.5 promoter by approximately 70% (p ≤ 0.0001; Fig. 2).

## Discussion

Herpesviruses have evolved to establish a life-long latency in their hosts, characterised by periodic reactivations resulting in the production of infectious virus. In the latent state, the virus genome exists as an episome, and expresses only a few genes. Initiation of the productive cycle is associated with an alteration in the genomic architecture that is accompanied by the induction of a temporal cascade of viral gene transcription, promoting viral genome replication and particle formation (Poudyal et al. 2017).

In vivo, shedding of OvHV-2 in nasal secretions from both adolescent and adult sheep occurs in short, sharp bursts (Li et al. 2004). Results of analysing the structure of the OvHV-2 genome in lymphoblasts derived from sheep are consistent with the virus being in a latent state; the genome was found to be episomal and a restricted pattern of viral gene expression was observed in these cells, with only ORF73 and Ov3.5 reliably detected (Thonur et al. 2006). Thus, in vivo and in vitro evidence suggests that OvHV-2 within the reservoir species exhibits tight control of latency and the reactivation process. In contrast, to the reservoir species, OvHV-2 structural proteins have been detected within lesions of SA-MCF affected animals, suggesting some level of lytic replication, aberrant or otherwise, occurs in the tissues of SA-MCF affected animals (Cunha et al. 2012). Moreover, lymphoblasts, derived from infected cattle, show the conformation of the OvHV-2 genome to be a mixture of linear and circular, with a wide range of genes detectable (Thonur et al. 2006). These observations, indicate that regulation of the OvHV-2 lytic and latent replication cycles may be less stringent in MCF susceptible animals compared to that which occurs in sheep, the natural host.

In EBV, expression of the virally encoded b-ZIP protein BZLF1 is sufficient to reactivate the virus from latency, by stimulating expression of BRLF1, the EBV homolog of RTA (reviewed by (Murata 2014)). In addition, a further virus protein, BRRF1, has been shown to function, in conjunction with BRLF1, to stimulate the lytic cycle (Hong et al. 2004). In this study, we examined the ability of the bZIP protein encoded by OvHV-2, Ov2, to stimulate expression of RTA, encoded by ORF50, and of the BRRF1 homolog ORF49. In the reporter system we used, Ov2 was unable to stimulate transcription from either the ORF50 or ORF49 promoters. It is therefore unlikely that by itself, Ov2, would be sufficient to reactivate OvHV-2 from latency and is therefore not a functional homolog of BZLF1.

In contrast, to Ov2, the OvHV-2 RTA in our reporter system was able to stimulate itself (ORF50) and viral genes associated with DNA replication (ORF6) and particle formation (ORF25). These results suggest that the RTA of OvHV-2 may,like those of MHV-68 (Wu et al. 2000) and KSHV (Gradoville et al. 2000), be sufficient, by itself, to drive entry of the virus into the lytic cycle.

Our results did provide evidence that Ov2 functions as a modulator of RTA, and our observations that Ov2 modulates RTA activity to repress transcriptional activation of the ORF57 and ORF50 promoters are consistent with those previously observed for the b-ZIP protein of KSHV, K8 (Izumiya et al. 2003; Lefort and Flamand 2009). Moreover, in MHV-68, the homolog of BRRF1 is a constituent part of the MHV-68 virion, and has been demonstrated to be required for optimal lytic replication of the virus in vitro and in vivo (Noh et al. 2012). The requirement of Ov2, in our system, to induce RTA into stimulating transcription of ORF49, the OvHV-2 homolog of BRRF1 (Hart et al. 2007), suggests that in vivo expression of Ov2 may be required for optimal OvHV-2 lytic replication*.* That Ov2 does not alter RTA-mediated expression of the early gene ORF6 or the late gene ORF25 supports the argument that the observed modulations of RTA, within our system, are specific and that Ov2 functions early in infection.

Evidence from other gamma herpesviruses suggests that vBcl-2 proteins have a role to play in maintaining the chronic infection of hosts (Coleman et al. 2014; E et al., 2009). OvHV-2, like EBV, encodes two Bcl-2 gene homologs (Hart et al. 2007) and we show that Ov2 coordinates with RTA to differentially regulate expression of these encoded viral Bcl-2 homologs, Ov4.5 and Ov9. The observation of differential regulation of the OvHV-2 Bcl-2 gene homologs is consistent with a previous observation that showed differential effects on the levels of expression of Ov4.5 and Ov9 in latently infected LGLs derived from SA-MCF affected cattle, when these cells were treated in vitro with 5-Azacytidine, a hypomethylating agent (Thonur et al. 2006). Ov9 contains a solitary Bcl-2 Homology 1 (BH1) domain with an NWGR motif shown to be important for protecting cells against apoptosis (Yin et al. 1994), suggesting it functions as an antagonist of apoptosis. Further support for this view, comes from the observation that A9 of AlHV-1, which shares significant homology with Ov9 at the amino acid level, protects cells against cisplatin induced apoptosis in vitro (Stowe 2005).

Ov2 shares significant homology, around the b-ZIP domain with A2, its positional homolog in AlHV-1 (Parameswaran et al. 2014). The observation made by Parameswaran et al that apoptotic pathways are upregulated in rabbit LGL cells derived from rabbits infected with an A2 deletant virus, compared to controls is consistent with a model in which A9 expression is regulated through A2 (Parameswaran et al. 2014). The availability of a bacterial artificial chromosome of AlHV-1, may prove a valuable tool in delineating to what extent the functions of Ov2/A2, and those of Ov9/A9, are conserved (Dewals et al. 2006).

Transcription of the Ov2 promoter is upregulated less than two fold in the presence of the OvHV-2 RTA. In addition, at least in the BHK21 cell-line used in this study, no positive feedback loop mediated via RTA, in conjunction with either ORF49 or Ov2, could be detected (data not shown). This may indicate that Ov2 is only required at low levels to fulfil its function within OvHV-2 biology or it may suggest that optimal Ov2 expression occurs in response to cellular signals and/or requires a cellular factor or factors expressed only in specific cell-types, for example in T cells that were absent from this experimental system. This would reflect the situation in KSHV where, activation of the B cell receptor has been demonstrated to trigger virus reactivation (Kati et al. 2013) and also MAPK cascade regulation of c-Jun and c-Fos expression (Reviewed by (Whitmarsh 2007). we have previously shown that OvHV-2 encodes a miRNA, ovhv2-miR-5, which functions to regulate the expression of RTA (Riaz et al. 2014). The accompanying paper (Nightingale et al. 2019) reports that two OvHV-2 encoded miRNAs can regulate expression of Ov2, adding a further layer of complexity to control of OvHV-2 gene expression. Further research into the different cellular mechanisms which trigger/repress the expression of the viral transactivators, such as Ov2, between SA-MCF susceptible animals and the reservoir host may offer significant insight into observed dysregulation in OvHV-2 replication seen in MCF affected animals, and the lymphoblasts derived from them.

## Conclusion

In this study, Ov2 is shown to act both as a transactivator and transrepressor of RTA-mediated virus gene expression. In particular, Ov2 induces expression of ORF49 and Ov9 that are likely candidates for proteins required for reactivation of the virus. It is therefore likely that Ov2 acts as a functional homolog to KSHV K8.
